# Burnout among elementary and high school teachers in three Canadian provinces: prevalence and predictors

**DOI:** 10.3389/fpubh.2024.1396461

**Published:** 2024-04-26

**Authors:** Belinda Agyapong, Raquel da Luz Dias, Yifeng Wei, Vincent Israel Opoku Agyapong

**Affiliations:** ^1^Department of Psychiatry, Faculty of Medicine & Dentistry, University of Alberta, Edmonton, AB, Canada; ^2^Department of Psychiatry, Faculty of Medicine, Dalhousie University, Halifax, NS, Canada

**Keywords:** burnout, depersonalization, emotional exhaustion, lack of accomplishment, resilience, stress, Wellness4Teachers, prevalence

## Abstract

**Background:**

Burnout is a longstanding issue among educators and has been associated with psychological and physical health problems such as depression, and insomnia.

**Objective:**

To assess the prevalence and predictors of the three dimensions of burnout (emotional exhaustion, depersonalization and lack of professional accomplishment) among elementary and high school teachers.

**Methods:**

This is a quantitative cross-sectional study with data collected via an online survey. The Maslach Burnout Inventory-Educator Survey (MBI-ES), the Brief Resilience Scale (BRS) and the Perceived Stress Scale were used, respectively, to assess burnout, resilience and stress among teachers. Data was collected between September 1st, 2022 and August 30th, 2023. SPSS (version 28, IBM Corp) was used for the data analysis.

**Results:**

Overall, 1912 educators received a link to the online survey via a text message, and 780 completed the burnout survey questions, resulting in a response rate of 41%. The prevalence of emotional exhaustion, depersonalization, and lack of professional accomplishment were 76.9, 23.2, and 30.8%, respectively. Participants with high-stress symptoms were 6.88 times more likely to experience emotional exhaustion (OR = 6.88; 95% CI: 3.31–14.29), 2.55 times (OR = 2.55; 95% CI: 1.65–3.93) more likely to experience depersonalization and 2.34 times (OR = 2.34; 95% CI: 1.64–3.35) more likely to experience lack of professional fulfilment. Additionally, respondents with low resilience were 3.26 times more likely to experience emotional exhaustion symptoms (OR = 3.26; 95% CI: 2.00–5.31), than those with high resilience. Males were about 2.4 times more likely to present with depersonalization compared to female teachers, whilst those who indicated their marital status as partnered or cohabiting and those who selected “other” were 3.5 and 7.3 times, respectively, more likely to present with depersonalization compared with those who were single. Finally, Physical Education were 3.8 times more likely to present with depersonalization compared with English teachers.

**Conclusion:**

The current study highlights the predictive effects of low resilience and high stress on the three dimensions of burnout among teachers in Canada. Interventions aimed at addressing systemic stress and fostering resilience are needed to reduce burnout among teachers.

## 1 Introduction

Burnout is a work-related dysphoria which occurs particularly among human service occupations where a high level of interpersonal engagements or human relationships may lead to chronic stress (1). Chronic stress has been associated with burnout, defined by three dimensions of overpowering exhaustion (emotional exhaustion), cynicism (feelings of cynicism and detachment from work), and inefficacy (a sense of ineffectiveness and lack of accomplishment) which conceptualize the individual stressful experience in a social context (1, 2). Burnout has also been defined as the state of exhaustion or loss of energy that occurs when individuals are incapable of meeting the high demands caused by their excessive workloads (3, 4). Burnout is a longstanding issue among educators, with a reported prevalence of moderate to severe burnout ranging from 25.12 to 74% (5). In a meta-analysis which included nine studies from eight different countries in Africa, Asia, Europe, and North and South America, the pooled prevalence of burnout among teachers during the COVID-19 pandemic was 52% (95% CI 33–71%), which is higher than burnout rates reported for health professionals (6). Chronic stress and burnout have been associated with poor physical and mental wellbeing and may lead to conditions such as depression, insomnia, diabetes, coronary heart disease, gastrointestinal issues, respiratory problems, and mortality below the age of 45 years (7). Burnout is a common phenomenon among teachers who often become vulnerable and succumb to the stressors they experience at work (8). In a recent cross-sectional study, 33.3% of teachers reported high burnout symptoms (9). In the lack of personal accomplishment dimension, one study reported a prevalence of 28.43% high burnout among teachers (10). Another study reported that 49.6% of teachers experience moderate or severe emotional exhaustion, 28.7% experience depersonalization, and 54.1% experience inefficacy (11). A systematic review and meta-analysis of studies assessing burnout among physical education teachers (12) reported a prevalence rate of 28.6% for high emotional exhaustion, 14.5% for depersonalization and 29.5% for low personal accomplishment. Another cross-sectional study (13) reported that emotional exhaustion had a significant direct effect on job satisfaction and turnover intentions.

With respect to teacher burnout, relationship quality has been cited to be a significant predictor of emotional exhaustion (14), and this highlights the importance of human relationships and collegiality among teachers as an important antidote for addressing the problem of stress and burnout. This was reiterated in a study which reported that educators’ level of burnout was positively correlated with burnout among those in their support networks (15). Similarly, a study among nurses reported that burnout could be ‘infectious, and for instance, in the dimension of depersonalization and emotional exhaustion, the complaints of perceived burnout among colleagues were the most significant predictor of burnout at the personal level and work environment (16). Both individual and environmental factors contribute to the development of burnout among teachers. A study reported that individual variables such as teaching efficacy, internal rewards and environmental features like support from principals and peers had a strong association with teacher reports of burnout (17). Another study reported that teachers’ efficacy in their ability to perform their job effectively was accountable for 82% of changes in burnout (18). Work overload has also been reported to give rise to burnout by reducing the ability of individuals to meet job demands, rest, recuperate, and restore balance, particularly when it’s chronic (1). This may lead to poor work performance, negative interaction with colleagues and students, low productivity, poor mental health, feelings of incompetence and poor quality of life (11). Resilience, characterized by one’s ability to adapt positively to adversity and maintain psychological wellbeing in the face of challenges (5, 19), appears as a robust predictor across all dimensions of burnout. Individuals with high resilience have less tendency to be burnt out and have an increased ability to easily bounce back after stressful mishaps. The behavior patterns of students have been reported to contribute to predicting burnout among teachers, with male teachers’ burnout primarily affected by students’ inattentiveness, while students’ disrespectful behavior mainly causes burnout among female teachers (20). Educational situations such as classroom conflict can have a negative impact on teachers’ levels of stress and burnout. One study noted that Cultivating Awareness and Resilience in Education (CARE), a professional development program had a statistically significant positive effect on the emotional support domain of the class, promoted teachers’ social and emotional competence and increased the quality of teachers’ classroom interactions (21). A study among teachers in Canada noted that increased co-worker support was associated with the prediction of burnout, predominantly increased feelings of accomplishment and decreased depersonalization (22). Conflicting outcomes are reported in the literature with regards to the labor demands that affect each of the educational stages. One study during the pandemic that compare burnout syndrome indicators at different levels of teaching reported that basic education teachers had higher burnout rate scores than higher education teacher (23). Dealing with problematic students was perceived as the main stressor for majority of elementary school teachers in another study (24). This study also suggested that irrespective of school type, quantitative workload and long working hours were the most significant factors affecting teachers’ stress responses.

Sociodemographic and professional factors such as age, sex, marital status, relationship quality, and teaching experience have also been associated with burnout in the literature (5, 25). A cross-sectional study also noted an association between years of teaching and increased burnout, with younger teachers with fewer years of experience reporting higher levels of emotional exhaustion and disengagement from the profession due to burnout (26). Another study among teachers reported higher scores on Emotional Exhaustion and Depersonalization among Canadian teachers than their Dutch peers and sex and type of school appeared significantly related to burnout across the two countries (27). Burnout has a significant positive predictive effect and correlation with turnover (28). Teachers leaving the profession due to burnout is problematic since teaching requires specific training and skill, and individuals with no previous teaching history will find it difficult to step into the role of teachers with long-term experience (29). To the best of our knowledge, there is no published literature on teachers stress and burnout specific to Alberta, Nova Scotia and Newfoundland and Labrador. Thus, this study provides a broader contextualization of the educational situation in Canada particularly the three provinces with respect to the prevalence of the three-burnout syndromes. This study therefore seeks to bridge the literature gap in this area.

Considering all the aforementioned, the current study aims to explore the prevalence and predictors of burnout (emotional exhaustion, depersonalization and lack of accomplishment) in the Canadian context, focusing on three participating provinces: Alberta, Nova Scotia and Newfoundland and Labrador. The specific objectives of this study include:

- To estimate the prevalence of emotional exhaustion, depersonalization and lack of accomplishment as measured with the Maslach Burnout Inventory among educators in Alberta, Nova Scotia and Newfoundland and Labrador.
- To determine the predictors of emotional exhaustion, depersonalization and lack of accomplishment as measured among educators in Alberta, Nova Scotia and Newfoundland and Labrador.

Presently, it is unknown what the prevalence and correlates of burnout are among teachers in the three provinces. The current research will, therefore, add to the literature, encourage more research in this field and further develop appropriate interventions to support teachers’ mental health and wellbeing. It is hypothesized that the prevalence of burnout among teachers in the three Canadian provinces would be comparable to those reported in a scoping review among teachers in other jurisdictions (5). It is also hypothesized that demographic characteristics such as age and gender, and professional related factors such as class size and years of teaching will predict each of the three burnout syndromes among the teachers as identified in the scoping review (5).

## 2 Methodology

### 2.1 Study design

This is a quantitative cross-sectional study design. Data was collected using web-based administered questionnaires with the University of Alberta REDCap platform (30). REDCap is a secure web application for the construction and management of surveys and data.

### 2.2 Ethics approval

Approval for the study was granted by the University of Alberta Ethics Review Board (Pro00117558) and the Dalhousie University Human Research Ethics Review Board (REB # 2023–6,840). Participants had access to the information leaflet accompanying the survey questions. Consent was obtained from all subjects.

### 2.3 Study settings

The study was conducted in the Canadian provinces of Alberta, Nova Scotia, Newfoundland and Labrador. Alberta, a Western Canada province, has an estimated population of 4,756,408 and approximately 32,523 full and part-time teachers (31, 32). Newfoundland and Labrador and Nova Scotia are both eastern provinces of Canada, with an estimated population of 540,418 and 1,066,416, respectively, (32). The English School District school board in Newfoundland and Labrador oversees all English-language primary and secondary education in the province, with more than 250 schools, approximately 63,000 students, and over 10,000 employees (33). The Nova Scotia Teachers Union represents more than 10,000 public school teachers, Community College faculty and professional support staff and teachers (34). The average secondary school class size in Canada is 26.4 which is slightly higher than the OECD average of 26.1 (35). The average class size is about 25 in Newfoundland and Labrador, 27 in Nova Scotia and 28.5 in Alberta (35). The salary range for elementary and high school teachers in the three provinces is as follows: Alberta- $59,488- $94,103, Nova Scotia- $56,268-$89,296, Newfoundland and Labrador- $55,932-$95,970 (36). The average number of years from starting a teaching career to reaching the top salary in Alberta, Nova Scotia and Newfoundland and Labrador are 10, 9, and 8 years respectively (36).

### 2.4 Data collection and outcome measures

### 2.5 Data collection

Data was collected between September 1st 2022, and August 30th 2023, via the Wellness4Teachers program. The Wellness4Teachers program (37) is a self-subscription, unidirectional program delivering daily supportive cognitive behavioral-based text messages to teachers. The program was launched and promoted among teachers in Alberta, Nova Scotia and Newfoundland and Labrador at the onset of the 2022/2023 academic year. Teachers in the three provinces could subscribe to the Wellness4Teachers program by texting “TeachWell” to a designated phone number. Teachers who subscribed were requested to complete the baseline surveys through a link delivered by text as part of the initial welcome message. Completing the baseline survey was voluntary and independent of receipt of the daily supportive text messages. The web-based questionnaires collected sociodemographic including sex, age, marital, ethnicity, housing status, professional (class size, number of years teaching, area of specialization, major role, major source of stress), and clinical (Burnout- emotional exhaustion, depersonalization and lack of accomplishment), stress and resilience variables. Typically, 5–10 min is required to complete the survey questionnaire.

### 2.6 Outcome measures

The Maslach Burnout Inventory-Educator Survey (MBI-ES) (38, 39) was used to collect data on burnout levels among teachers. The MBI-ES contains a 22-item instrument that measures emotional exhaustion, depersonalization, and personal accomplishment. A 7-point Likert scale is used to determine and indicate the frequency with which educators agree with the statements: 0 (never); 1 (a few times a year or less); 2 (once a month or less); 3 (a few times a month); 4 (once a week); 5 (a few times a week) 6 (every day). Questions on the MBI-ES include:

1. _________ I feel emotionally drained from my work.
2. _________ I feel used up at the end of the workday.
3. _________ I feel fatigued when I get up in the morning and have to face another day on the job.
4. _________ I can easily understand how my students feel about things.
5. _________ I feel I treat some students as if they were impersonal objects.
6. _________ Working with people all day is really a strain for me.
7. _________ I deal very effectively with the problems of my students.
8. _________ I feel burned out from my work.
9. _________ I feel I’m positively influencing other people’s lives through my work.
10. ________ I’ve become more callous toward people since I took this job.
11. _________I worry that this job is hardening me emotionally.
12. _________I feel very energetic.
13. _________I feel frustrated by my job.
14. _________I feel I’m working too hard on my job.
15. _________I do not really care what happens to some students.
16. _________Working with people directly puts too much stress on me.
17. _________I can easily create a relaxed atmosphere with my students.
18. ________ I feel exhilarated after working closely with my students.
19. _________I have accomplished many worthwhile things in this job.
20. _________I feel like I’m at the end of my rope.
21. ________ In my work, I deal with emotional problems very calmly.
22. _________ I feel students blame me for some of their problems.

Emotional exhaustion is assessed by summing responses to items numbers 1, 2, 3, 6, 8, 13, 14, 16, and 20. Depersonalization is assessed by summing survey responses to items numbers 5, 10, 11, 15, and 22, and personal accomplishment is assessed by summing survey responses to item numbers 4, 7, 9, 12, 17, 18, 19, and 21. For each of the three dimensions, emotional exhaustion scores ≥27 were high, 17–26 were normal, and 0–16 was low. Similarly, for depersonalization scores, 13 and over was high, 7–12 was normal, and 0–6 was low. Finally, for personal accomplishment, 0–31 was high, 32–38 was normal, and 39 and over was low. An analysis for internal consistency of the MBI-ES questionnaire yielded a Cronbach’s alpha of 0.785 for the full scale, which indicated scale reliability. The alpha coefficient for the emotional exhaustion subscale was 0.93 suggesting excellent reliability, while the alpha coefficient for the depersonalization subscale was 0.618 suggesting questionable reliability and the alpha coefficient for the personal accomplishment subscale was 0.776 indicating acceptable reliability (40). In a confirmatory factor analysis to test the data fit to the original three-factor structure proposed for the MBI using a sample of health professionals, the chi-square ratio by the degrees of freedom (*χ*^2^/df) ranged between 1.86 and 2.16, the comparative fit index (CFI) ranged between 0.86 and 0.93, the goodness of fit index (GFI) ranged between 0.86 and 0.90, the Tucker Lewis index (TLI) ranged between 0.85 and 0.91 and the root mean square error of approximation (RMSEA) ranged between 0.06 and 0.07 (41). The model derived after removing items 9 and 15 and had indexes *χ*^2^/df = 1.84; CFI = 0.93; GFI = 0.90; TLI = 0.91 and RMSEA = 0.06, was considered the best in relation to the three models.

The Brief Resilience Scale (BRS; mean scores of 1.00 to 2.99 suggests low resilience, 3.00 to 4.30 suggest normal resilience, and 4.31 to 5.00 suggest high resilience) (42), was used to assess resilience and stress was measured using the Perceived Stress Scale 10 (PSS-10; score of ≤26 indicates low stress, a score of ≥27 indicates likely high stress) (43). The BRS and the PSS-10 have Cronbach’s *α* of 0.78 and 0.82 respectively, suggesting good internal consistency (44, 45). In a study (46) to investigate the two-factor structure of the Korean version of the BRS, the Chi-square statistic (*χ*^2^ = 391.92, *χ*^2^/df = 48.99) was significant. However, the SRMR (0.01), RMSEA (0.06), GFI (0.99), and CFI (0.99) were well within the limits that indicate model acceptability, demonstrating that the model with two first-order components for questions that were positively and negatively worded offered a good fit to the BRS scores. In a systematic review and meta-analytic confirmatory factor analysis of the PSS-10 (47), it was decided that the most appropriate factor structure of the PSS-10 was the correlated two-factor model with indexes: CFI = 0.998; TLI = 0.998; RMSEA = 0.013, 90% CI [0.012, 0.015]; standardized root mean square residual (SRMR) = 0.011.

The MBI-ES, BRS and PSS-10 scales were studied as categorical variables for the purpose of prevalence estimates. The primary outcome measures were the prevalence of high emotional exhaustion, high depersonalization and low personal accomplishment at baseline in subscribers of the Wellness4Teachers program. Secondary outcome measures included professional, sociodemographic, and clinical correlates of these three dimensions of burnout.

### 2.7 Statistical analysis

SPSS (version 28, IBM Corp.) (48) was used for the data analysis. Descriptive statistics were summarized for demographic, professional and clinical variables, as well as prevalence estimates based on the province in which the teachers are located. The Chi-square/Fisher’s exact test was employed to determine the relationship between each of the demographic, clinical, and professional variables. Percentages and numbers were used to report the descriptive characteristics, and a 2-tailed *p* ≤ 0.05 was used to determine statistical significance for all analyses. Three independent binary logistic regression analyses were used to identify variables that were independently predictive of likely high emotional exhaustion, high depersonalization and low personal accomplishment among participants. Only variables which had a significant (*p* < 0.05) or had a trend towards significant (0.1 ≥ *p* ≥ 0.05) relationship with likely high emotional exhaustion, high depersonalization and low personal accomplishment in the Chi-square/Fisher’s exact tests were included in the regression models. Surveys with more than 50% missing responses were omitted from data analysis, and results are based only on completed survey data with no imputation of missing data (37).

## 3 Results

Table 1 below gives a summary of the distribution of demographic, work-related and professional variables based on participants’ province of residence (Alberta, Nova Scotia, Newfoundland and Labrador). Overall, 1,912 participants enrolled in the Wellness4Teachers program and received the link and invitation to complete the baseline web-based survey. Out of this, 780 completed all the demographic and professional questions, the Maslach Burnout Inventory, the Perceived Stress Scale and the Brief Resilience Scale, resulting in a response rate of 40.79%. Of the 780 participants, 547 (70.1%) were in Alberta, 131 (16.8%) were residents in Nova Scotia, and 102 (13.1%) were residents in Newfoundland and Labrador.

**Table 1:** Frequency distribution of demographic, work-related and professional variables based on participants’ province (Alberta, Nova Scotia or Newfoundland and Labrador).

Variables	Alberta *n* (%) *N*=547	Nova Scotia *n* (%) *N*=131	Newfoundland *n* (%) *N*=102	Chi^2^/Fishers Exact^*^ Test Values	*p-*value	Total *n* (%) *N*=780
Sociodemographic characteristics						
Age (Years)						
18–25	20 (3.7%)	4 (3.1%)	2 (2.2%)	3.13	0.79	26 (3.3%)
26–40	206 (37.7%)	55 (42.0%)	33 (32.4%)			294 (37.7%)
41–60	306 (55.9%)	69 (52.7%)	64 (62.7%)			439 (56.3%)
61 and above	15 (2.7%)	3 (2.3%)	3 (2.9%)			21 (2.7%)
Sex at birth						
Male	66 (12.1%)	18 (13.7%)	12 (11.8%)	0.31	0.86	96 (12.3%)
Female	481 (87.9%)	113 (86.3%)	90 (88.2%)			684 (87.7%)
Relationship status						
Single	83 (15.5%)	21 (16.0%)	12 (11.8%)			116 (14.9%)
Married	361 (66.0%)	74 (56.5%)	65 (63.7%)	15.07	0.27	500 (64.1%)
Common- Law or Partnered	63 (11.5%)	29 (22.1%)	16 (15.7%)			108 (13.8%)
Separated or Divorced	35 (6.4%)	6 (4.6%)	6 (5.9%)			47 (6.0%)
Other	5 (0.9%)	1 (0.8%)	3 (2.9%)			9 (1.2%)
Number of children						
No child	175 (32.0%)	45 (34.4%)	23 (22.5%)			243 (31.2%)
One child	76 (13.6%)	17 (13.0%)	22 (21.6%)	10.35	0.24	115 (14.7%)
Two children	208 (38.0%)	48 (36.6%)	45 (44.1%)			301 (38.6%)
Three children	59 (10.8%)	16 (12.2%)	10 (9.8%)			85 (10.9%)
Four or more children	29 (5.3%)	5 (3.8%)	2 (2.0%)			36 (4.6%)
Ethnicity						
Indigenous	10 (1.8%)	5 (3.8%)	2 (2.0%)			17 (2.2%)
African Descendants	7 (1.3%)	0 (0.0%)	0 (0.0%)			7 (0.9%)
East Asian	12 (2.2%)	0 (0.0%)	0 (0.0%)			12 (1.5%)
Latino	8 (1.5%)	1 (0.8%)	0 (0.0%)			9 (1.2%)
Middle Eastern	5 (0.9%)	0 (0.0%)	0 (0.0%)	^*^12.38	0.45	5 (0.6%)
South Asian	8 (1.5%)	0 (0.0%)	0 (0.0%)			8 (1.0%)
Caucasian (European descent)	487 (89.0%)	123 (93.9%)	98 (96.1%)			708 (90.8%)
Other ethnicities	(10 1.8%)	2 (1.5%)	2 (2.0%)			14 (1.8%)
Housing status						
Own home	458 (83.7%)	104 (79.4%)	85 (83.3%)	^*^2.02	0.48	647 (82.9%)
Rented accommodation.	75 (13.7%)	20 (15.5%)	15 (14.7%)			110 (14.1%)
Live with family or friend	14 (2.6%)	7 (5.3%)	2 (2.0%)			23 (2.9%)
Professional variables						
Location of school						
Rural setting	168 (30.7%)	78 (59.5%)	55 (53.9%)	48.71	0.000	301 (38.6%)
Urban setting	379 (69.3%)	53 (40.5%)	47 (46.1%)			479 (61.4%)
Area of teaching specialization						
English	95 (17.4%)	19 (14.5%)	13 (12.7%)			127 (16.3%)
Mathematics	36 (6.6%)	13 (9.9%)	8 (7.8%)	^*^22.2	0.03	57 (7.3%)
Sciences (Physics, Chemistry, Biology)	35 (6.4%)	4 (3.1%)	15 (14.7%)			54 (6.9%)
Arts (History, Geography, Social Studies, etc.)	55 (10.1%)	8 (6.1%)	8 (7.8%)			71 (9.1%)
Music	18 (3.3%)	2 (1.5%)	(3 2.9%)			23 (2.9%)
Physical education	17 (3.1%)	4 (3.1%)	0 (0.0%)			21 (2.7%)
Other	291 (53.2%)	81 (61.8%)	55 (53.9%)			427 (54.7%)
Teach only in your area of specialization.						
No	338 (61.8%)	62 (42.3%)	48 (47.1)	14.2	**0.001**	448 (57.4%)
Yes	209 (38.2%)	69 (52.7%)	54 (52.9%)			332 (42.6%)
Number of years teaching						
5 years or less	78 (14.3%)	25 (19.1%)	8 (7.8%)			111 (14.2%)
10 years or less but more than 5 years	109 (19.9%)	14 (10.7%)	16 (15.7%)	16.61	**0.01**	139 (17.8%)
20 years or less but more than 10 years	206 (37.7%)	56 (42.7%)	36 (35.3%)			298 (38.2%)
More than 20 years.	154 (28.2%)	36 (27.5%)	42 (41.2%)			232 (29.7%)
Average number of students/pupils in the classes you teach						
20 or less	91 (16.6%)	33 (25.2%)	40 (39.2%)			164 (21.0%)
21–27	288 (52.7%)	79 (60.3%)	39 (38.2%)	38.79	**0.00**	406 (52.1%)
28 or more	168 (30.7%)	19 (14.5%)	23 (22.5%)			210 (26.9%)
School you teach						
Catholic School	141 (25.8%)	0 (0.0%)	1 (1.0%)	110.61	**0.000**	142 (18.2%)
Public School	398 (72.8%)	131 (100.0%)	93 (91.2)			622 (79.7%)
Other	8 (1.5%)	0 (0.0%)	8 (7.8%)			16 (2.1%)
Major Role						
Elementary school teacher	254 (46.4%)	64 (48.9%)	26 (25.5%)			344 (44.1%)
Junior high school teacher	94 (17.2%)	23 (17.6%)	24 (23.5%)	43.82	**0.000**	141 (18.1%)
Senior high school teacher	80 (14.6%)	22 (16.8%)	13 (12.7%)			115 (14.7%)
Support Staff	20 (3.7%)	10 (7.6%)	3 (2.9%)			33 (4.2%)
Administrator	54 (9.9%)	2 (1.5%)	14 (13.7%)			70 (9.0%)
Other	45 (8.2%)	10 (7.6%)	22 (21.6%)			77 (9.9%)
Source of stress						
Workload	314 (57.4%)	76 (58.0%)	45 (44.1%)			435 (55.8%)
Student Behavior	101 (18.5%)	33 (25.2%)	29 (28.4%)	21.12	**0.007**	163 (20.9%)
Class size	29 (5.5%)	2 (1.5%)	11 (10.8%)			42 (5.4%)
Lack of support from school administration	47 (8.6%)	9 (6.6%)	4 (3.9%)			60 (7.7%)
Other	56 (10.2%)	11 (8.4%)	13 (12.7%)			80 (10.3%)
Stress, resilience, and burnout variables						
High stress						
No	393 (71.8%)	97 (74.0%)	82 (80.4%)	3.25	0.2	572 (73.3%)
Yes	154 (28.2%)	34 (26.0%)	20 (19.6%)			208 (26.7%)
Low resilience						
No	309 (58.6%)	75 (59.5%)	66 (67.3%)	2.62	0.27	450 (59.9%)
Yes	201 (41.4%)	51 (40.5%)	32 (32.7%)			301 (40.1%)
Emotional Exhaustion						
No	123 (22.5%)	22 (16.9%)	35 (34.3%)	10.10	**0.006**	180 (23.1%)
Yes	423 (77.5%)	108 (83.1%)	67 (65.7%)			598 (76.9%)
Depersonalization						
No	425 (75.3%)	108 (82.4%)	79 (77.5%)	3.04	0.22	599 (76.8%)
Yes	135 (24.7%)	23 (17.6%)	23 (22.5%)			181 (23.2%)
Lack of Professional Accomplishment						
No	378 (69.1%)	85 (65.4%)	76 (74.5%)	2.24	0.34	539 (69.2%)
Yes	169 (30.9%)	45 (34.6%)	26 (25.5%)			240 (30.8%)

The majority of the participants were aged between 41 and 60 (439, 56.3%); most were Caucasians (708, 90.8%), female (684, 87.7%), married (500, 64.1%), and less than half had two children (301, 38.6%). Significantly more participants live in the urban setting than rural setting (479, 61.4%), are elementary school teachers (344, 44.1%), teach in a public school (622 79.7%), teach in areas outside their specialization (448 57.4%), have been teaching for 20 years or less but more than 10 years (298, 38.2%), have an average of 21 to 27 students in the classes they teach (406, 52.1%), and 55.8% (435) identified workload as their source of stress. Regarding the clinical variables, the prevalence of high stress was 26.7% (208), low resilience was 40.1% (301), emotional exhaustion was 76.9% (598), depersonalization was 181 (23.2%), and lack of professional accomplishment was 30.8% (240).

### 3.1 Univariate analysis

Table 2 gives the results of Chi-square/Fisher’s exact test of association between demographic, professional and clinical characteristics, and emotional exhaustion, depersonalization and personal fulfilment. Eleven variables had a statistically significant (*p* < 0.05) or trend towards significant (0.1 ≥ *p* ≥ 0.05) association with emotional exhaustion. These variables are age, province of teachers, number of children, ethnicity, housing status, where the school is situated, number of years teaching, kind of school teachers teach, major role, stress, and resilience. Participants aged 18–25 years who live in Nova Scotia, have no child, are of Middle Eastern origin, live with family or friends, teach in a school in an urban setting, junior high teachers with 10 years or less but more than 5 years teaching experience, teach in the public school, and have high stress and low resilience had emotional exhaustion relative to participants in their respective categories.

**Table 2:** Chi-square test of association between demographic, work-related, clinical, and burnout characteristics.

Variables	Emotional exhaustion absent *n* (%)	Emotional exhaustion present *n* (%)	Chi^2^/Fisher Exact	*P-*value	Depersonalization/Cynicism absent *n* (%)	Depersonalization/Cynicism present *n* (%)	Chi^2^/Fisher Exact	*P-*value	Professional fulfilment present *n* (%)	Professional fulfillment absent *n* (%)	Chi^2^/Fisher Exact	*P-*value
Sociodemographic characteristics												
Age (Years)												
18–25	4 (15.4%)	22 (84.6%)	21.90	**0.000**	19 (73.1%)	7 (26.9%)	15.57	**0.002**	13 (50.0%)	13 (50.0%)	8.43	**0.03**
26–40	51 (17.3%)	243 (82.7%)			204 (69.4%)	90 (30.6%)			194 (66.2%)	99 (33.8%)		
41–60	113 (25.9%)	324 (71.4%)			359 (81.8%)	80 (18.2%)			315 (71.8%)	124 (28.2%)		
61 and above	12 (57.1%)	9 (42.9%)			17 (81.0%)	4 (19.0%)			17 (81.0%)	4 (19.0%)		
Sex at birth												
Male	26 (27.1%)	70 (72.9%)	0.96	0.37	59 (61.5%)	37 (38.5%)	14.45	**0.000**	61 (63.5%)	35 (36.6%)	1.64	0.24
Female	154 (22.6%)	528 (77.4%)			540 (78.9%)	144 (21.1%)			478 (70.0%)	205 (30.0%)		
Provinces												
Alberta	123 (22.5%)	423 (77.5%)	10.10	**0.006**	412 (75.3%)	135 (24.7%)	3.04	0.22	378 (69.1%)	169 (30.9%)	2.24	0.33
Newfoundland	25 (34.3%)	67 (65.7%)			79 (77.5%)	23 (22.5%)			76 (74.5%)	26 (25.5%)		
Nova Scotia	22 (16.9%)	108 (83.1%)			108 (82.4%)	23 (17.6%)			85 (65.4%)	45 (34.6%)		
Relationship status												
Single	22 (19.0%)	94 (81.0%)			91 (78.4%)	25 (21.6%)			76 (66.1%)	39 (33.9%)		
Married	122 (24.4%)	377 (75.6%)	3.98	0.41	398 (79.6%)	102 (20.4%)	13.02	**0.009**	351 (70.2%)	149 (29.8%)	1.83^*^	0.76
Common Law/Partnered	20 (18.7%)	87 (81.3%)			70 (64.8%)	38 (35.2%)			71 (65.7%)	37 (34.3%)		
Separated/Divorced	14 (29.8%)	33 (70.2%)			35 (74.5%)	12 (25.5%)			34 (72.3%)	13 (27.7%)		
Other	2 (22.2%)	7 (77.8%)			5 (55.6%)	4 (44.4%)			7 (77.8%)	2 (22.2%)		
# of children												
No child	37 (15.2%)	206 (84.8%)			166 (68.3%)	77 (31.7%)	18.31	**0.001**	164 (67.8%)	78 (32.3%)	4.21	0.38
One child	29 (25.2%)	86 (74.8%)	18.15	**0.001**	86 (74.8%)	29 (25.2%)			73 (63.5%)	42 (36.5%)		
Two children	72 (24.1%)	227 (75.9%)			251 (83.4%)	50 (16.6%)			218 (72.4%)	83 (27.6%)		
Three children	29 (34.1%)	56 (65.9%)			66 (77.6%)	19 (22.4%)			61 (71.8%)	24 (28.2%)		
Four or more children	13 (36.1%)	23 (63.9%)			30 (83.3%)	6 (16.7%)			23 (63.9%)	13 (36.1%)		
Ethnicity												
Indigenous	7 (42.1%)	10 (58.8%)			11 (64.7%)	6 (35.3%)	19.44	**0.003**	7 (41.2%)	10 (58.8%)	11.73	0.11
African Descendants	3 (42.9%)	4 (57.1%)	11.47^*^	**0.08**	4 (57.1%)	3 (42.9%)			5 (71.4%)	2 (28.6%)		
East Asian	2 (16.7%)	10 (83.3%)			4 (33.3%)	8 (66.7%)			5 (41.7%)	7 (58.3%)		
Latino	5 (55.6%)	4 (44.4%)			8 (88.9%)	1 (11.1%)			6 (66.7%)	3 (33.3%)		
Middle Eastern	0 (0.0%)	5 (100%)			3 (60.0%)	2 (40.0%)			3 (60.0%)	2 (40.0%)		
South Asian	1 (12.5%)	7 (87.5%)			4 (50.0%)	4 (50.0%)			5 (62.5%)	3 (37.5%)		
Caucasian	158 (22.4%)	548 (77.6%)			553 (78.1%)	155 (21.9%)			499 (70.6%)	208 (29.4%)		
Other ethnicities	4 (28.6%)	10 (71.4%)			12 (85.7%)	2 (14.3%)			9 (64.3%)	5 (35.7%)		
Housing status												
Own home	159 (24.7%)	486 (75.3%)	4.99	**0.08**	510 (78.8%)	137 (21.2%)	9.54	**0.008**	456 (70.5%)	191 (29.5%)	3.04	0.22
Renting	18 (16.4%)	92 (83.6%)			72 (65.5%)	38 (34.5%)			68 (62.4%)	41 (37.6%)		
Live with family or friend	3 (13.0%)	20 (87.0%)			17 (73.9%)	6 (26.1%)			15 (65.2%)	8 (34.8%)		
School Situated												
Rural setting	80 (26.8%)	219 (73.2%)	3.58	**0.07**	245 (81.4%)	56 (18.6%)	5.82	**0.02**	206 (68.4%)	95 (31.6%)	0.13	0.75
Urban Setting	100 (20.9%)	379 (79.1%)			354 (73.9%)	125 (26.1%)			333 (69.7%)	145 (30.3%)		
Area of teaching specialization												
English	35 (19.7%)	102 (80.3%)	3.10	0.80	104 (81.9%)	23 (18.1%)	22.18	**0.001**	91 (71.4%)	36 (28.3%)	4.2	0.66
Mathematics	14 (24.6%)	43 (75.4%)			42 (73.7%)	15 (26.3%)			35 (61.4%)	22 (38.6%)		
Sciences (Physics, Chemistry, Biology)	15 (27.8%)	39 (72.2%)			35 (64.8%)	19 (35.2%)			37 (68.5%)	17 (31.5%)		
Arts (History, Geography, Social Studies etc.)	20 (28.2%)	51 (71.8%)			49 (69.0%)	22 (31.0%)			46 (64.8%)	25 (35.2%)		
Music	5 (21.7%)	18 (78.3%)			13 (56.5%)	10 (43.5%)			14 (60.9%)	9 (39.1%)		
Physical education	6 (28.6%)	15 (71.4%)			12 (57.1%)	9 (42.9%)			13 (65.0%)	7 (35.0%)		
Other	95 (22.4%)	330 (77.6%)			344 (80.6%)	83 (19.4%)			303 (71.0%)	124 (29.0%)		
Only teach in area of specialization												
No	99 (22.2%)	347 (77.8%)	0.52	0.49	344 (76.8%)	104 (23.2%)	0.00	1.0	314 (70.2%)	133 (29.8%)	0.55	0.47
Yes	81 (24.4%)	251 (75.6%)			255 (76.8%)	77 (23.2%)			225 (67.8%)	107 (32.2%)		
Number of years teaching												
5 years or less	19 (17.1%)	92 (82.3%)	9.47	**0.02**	76 (68.5%)	39 (31.5%)	13.26	**0.004**	69 (62.7%)	41 (37.3%)	12.77	**0.005**
10 years or less but more than 5 years	22 (15.8%)	117 (84.2%)			96 (69.1%)	43 (30.9%)			84 (60.4%)	55 (39.6%)		
20 years or less but more than 10 years	78 (26.2%)	220 (73.8%)			239 (80.2%)	59 (19.8%)			209 (70.1%)	89 (29.9%)		
More than 20 years.	61 (26.5%)	169 (73.5%)			188 (81.0%)	44 (19.0%)			177 (76.3%)	55 (23.7%)		
Average number of students/pupils in the classes you teach.												
20 or less	47 (28.7%)	117 (71.3%)			132 (80.5%)	32 (19.5%)			115 (70.1%)	49 (29.9%)		
21–27	88 (21.8%)	316 (78.2%)	3.57	0.17	319 (78.6%)	87 (21.4%)	6.68	**0.04**	282 (69.5%)	124 (51.7%)	0.23	0.89
28 or more	45 (21.4%)	165 (78.6%)			148 (70.5%)	62 (29.5%)			142 (67.9%)	142 (67.8%)		
School you teach.												
Catholic School	37 (26.1%)	105 (73.9%)	7.82	**0.02**	110 (77.5%)	32 (22.5%)	0.14^*^	0.96	93 (65.5%)	49 (34.5%)	2.57	0.30
Public School	135 (21.8%)	485 (78.2%)			476 (76.5%)	146 (23.5%)			437 (70.4%)	184 (29.6%)		
Other	8 (50.0%)	8 (50.0%)			13 (81.3%)	3 (18.8%)			9 (56.3%)	7 (43.8%)		
Major Role												
Elementary teacher	69 (20.1%)	274 (79.9%)			274 (79.7%)	70 (20.3%)	19.7	**0.001**	227 (66.0%)	117 (34.0%)	10.85	**0.054**
Junior high teacher	26 (18.4%)	115 (81.6%)	15.11	**0.01**	95 (67.4%)	46 (32.6%)			96 (68.6%)	44 (31.4%)		
Senior high teacher	25 (21.7%)	90 (78.3%)			78 (67.8%)	37 (32.2%)			76 (66.1%)	39 (33.9%)		
Support Staff	12 (36.4%)	21 (63.6%)			28 (84.8%)	5 (15.2%)			22 (66.7%)	11 (33.3%)		
Administrator	21 (30.4%)	48 (69.9%)			59 (84.3%)	11 (15.7%)			57 (81.4%)	13 (18.6%)		
Other	27 (35.1%)	50 (64.9%)			65 (84.4%)	12 (15.6%)			61 (79.2%)	16 (20.8%)		
Source of stress												
Workload	99 (22.9%)	334 (77.1%)			348 (80.0%)	87 (20.0%)	8.79	**0.07**	313 (72.0%)	122 (28.0%)	7.26	0.12
Student Behavior	31 (19.0%)	132 (81.0%)	3.99	0.41	115 (70.6%)	48 (29.4%)			100 (61.7%)	62 (68.3%)		
Class size	13 (31.0%)	29 (69.0%)			35 (83.3%)	7 (16.7%)			32 (76.2%)	10 (23.8%)		
Limited management support	15 (25.0%)	45 (75.0%)			43 (71.7%)	17 (28.3%)			39 (65.0%)	21 (35.0%)		
Other	22 (27.5%)	58 (72.5%)			58 (71.5%)	22 (27.5%)			55 (68.8%)	25n (31.2%)		
Stress												
Mild to moderate stress	170 (29.8%)	400 (70.2%)	53.63	**0.000**	470 (82.2%)	102 (17.8%)	34.75	**0.000**	433 (75.7%)	139 (24.3%)	47.77	**0.00**
High stress	10 (4.8%)	198 (95.2%)			129 (62.0%)	79 (38.0%)			106 (51.2%)	101 (48.8%)		
Resilience												
Normal to high resilience	142 (31.6%)	308 (68.4%)	49.29	0.000	366 (81.3%)	84 (18.7%)	14.98	**0.000**	341 (75.8%)	109 (24.2%)	26.10	**0.000**
Low resilience	29 (9.6%)	272 (90.4%)			208 (69.1%)	93 (30.9%)			175 (58.1%)	126 (41.9%)		

^*^Fisher Exact Value.

Similarly, 14 variables had a statistically significant (*p* < 0.05) or near significant relationship (0.1 ≥ *p* ≥ 0.05) with likely depersonalization, including age, sex at birth, relationship status, number of children, ethnicity, housing status, where school is situated, area of teaching specialization, number of years teaching, major role, average number of students/pupils in the classes, source of stress, stress, and resilience. Male participants 26–40 years, of East Asian origin, no child, renting, music teacher in an urban setting, with 5 years or less teaching experience and 28 or more students in the class, Junior high school teachers, with student behavior as a source of stress, experiencing high stress and have low resilience experience depersonalization compared to other participants in their respective categories.

Finally, five variables had a statistically significant (*p* < 0.05) or near significant relationship (0.1 ≥ *p* ≥ 0.05) with likely personal fulfilment. These are age, teaching experience, major role in the school, stress, and resilience. Participants aged 18–25 years who are elementary school teachers with 10 years or less but more than 5 years of teaching experience, with low resilience and high stress, experience a lack of professional fulfilment in comparison to other respondents in their corresponding categories.

### 3.2 Logistic regression to identify predictors of emotional exhaustion

The outline of the binary logistic regression analysis is shown in Table 3. Eleven predictors that had a significant association (*p* < 0.05) or a trend towards significant association (0.1 ≥ *p* ≥ 0.05) with emotional exhaustion in the univariate analysis were included in a binary logistic regression model. The regression model was statistically significant; *Χ*^2^ (df = 31; *n* = 751) = 157.24 *p* = 0.000 < 0.0005, suggesting that the model could differentiate between participants who were emotionally exhausted and those who were not. Nonetheless, the model only explained between 18.9% (Cox and Snell *R*^2^) and 28.7% (Nagelkerke *R*^2^) of the variance and correctly classified 79.5% of cases.

**Table 3:** Logistic regression model for emotional exhaustion.

Variables	*B*	S.E	Wald	df	Sig.	Odds Ratio	95% C.I. for odds ratio	
							Lower	Upper
Age (Years)								
18–25			3.888	3	0.274			
26–40	0.420	0.719	0.341	1	0.559	1.522	0.372	6.232
41–60	0.423	0.763	0.308	1	0.579	1.527	0.342	6.806
61 and above	−0.525	0.896	0.343	1	0.558	0.592	0.102	3.428
Provinces								
Alberta			7.459	2	**0.024**			
Newfoundland	−0.483	0.286	2.840	1	0.092	0.617	0.352	1.082
Nova Scotia	0.520	0.308	2.841	1	0.092	1.682	0.919	3.078
Ethnicity								
Indigenous	−0.694		9.859	7	0.197			
African Descendants	−0.288	1.219	0.324	1	0.569	0.500	0.046	5.452
East Asian	−1.215	1.060	0.074	1	0.786	0.750	0.094	5.985
Latino	20.256	0.998	1.481	1	0.224	0.297	0.042	2.100
Middle Eastern	0.916	16154.434	0.000	1	0.999	0.752	0.000	0.000
South Asian	0.620	1.267	0.523	1	0.470	2.499	0.209	29.927
Caucasian	−0.324	0.594	1.090	1	0.296	1.859	0.581	5.951
Other ethnicities		0.917	0.124	1	0.724	0.724	0.120	4.368
Housing status								
Own home			1.822	2	0.402			
Renting	0.370	0.371	0.993	1	0.319	1.448	0.699	2.997
Live with family or friend	0.870	0.837	1.080	1	0.299	2.387	0.463	12.315
# of children								
No child			9.377	4	0.052			
One child	−0.587	0.342	2.947	1	0.086	0.556	0.285	1.087
Two children	−0.360	0.289	1.549	1	0.213	0.698	0.396	1.230
Three children	−0.813	0.365	4.960	1	**0.026**	0.443	0.217	0.907
Four or more children	−1.263	0.503	6.311	1	**0.012**	0.283	0.106	0.758
School Situated								
Urban Setting	0.491	0.212	5.372	1	**0.020**	1.634	1.079	2.474
Number of years teaching								
5 years or less			1.977	3	0.577			
10 years or less but more than 5 years	−0.167	0.458	0.133	1	0.716	0.846	0.345	2.078
20 years or less but more than 10 years	−0.446	0.427	1.090	1	0.297	0.640	0.277	1.479
More than 20 years.	−0.215	0.471	0.209	1	0.648	0.806	0.320	2.030
Major Role								
Elementary teacher			0.934	5	0.968			
Junior high teacher	0.004	0.298	0.000	1	0.989	1.004	0.560	1.800
Senior high teacher	−0.079	0.300	0.070	1	0.792	0.924	0.513	1.663
Support Staff	−0.410	0.467	0.769	1	0.381	0.664	0.266	1.658
Administrator	−0.017	0.354	0.002	1	0.961	0.983	0.491	1.966
Other	−0.142	0.324	0.193	1	0.661	0.868	0.460	1.636
School you teach.								
Catholic School			5.541	2	0.063			
Public School	0.284	0.269	1.117	1	0.291	1.328	0.785	2.249
Other	−1.139	0.687	2.748	1	0.097	0.320	0.083	1.231
Stress								
High stress	1.928	0.373	26.683	1	**0.000**	6.875	3.308	14.287
Resilience								
Low resilience	1.180	0.250	22.369	1	**0.000**	3.255	1.996	5.309
Constant	−0.176	0.932	0.036	1	0.850	0.839		

S.E, standard error; B, beta; df, degree of freedom; Sig, significance; C.I, confidence interval.

Table 3 indicates that with a Wald of 26.68 and 22.37, respectively, stress and resilience as predictors of emotional exhaustion made the most significant unique contribution to the regression model. School location, stress and resilience independently predicted the presence of emotional exhaustion among participants. After controlling for all other variables in the regression model, participants who worked in the urban setting were 1.63 times more likely to experience emotional exhaustion than those in the rural setting (OR = 1.63; 95% CI: 1.08–2.47). Similarly, respondents with high stress symptoms were 6.88 times more likely to experience emotional exhaustion than those who did not (OR = 6.88; 95% CI: 3.31–14.29). Additionally, respondents with low resilience were 3.26 times more likely to experience emotional exhaustion symptoms than those who did not (OR = 3.26; 95% CI: 2.00–5.31).

Although the province in which the respondent resides as a variable made a unique statistically significant contribution to the model, respondents in Nova Scotia and Newfoundland and Labrador were not significantly different from respondents in Alberta in terms of the likelihood to experience emotional exhaustion. Number of children as a variable did not make a unique statistically significant contribution to the model. However, participants who had no children were 2.26 times (OR = 2.26; 95% CI: 1.10–4.61) more likely to experience emotional exhaustion than those with three children. Similarly, participants with no children were 3.53 times (OR = 3.53; 95% CI: 1.32–9.43) more likely to experience emotional exhaustion than those who had four or more children. Other demographic and professional variables, such as age, ethnicity, housing status, major role, the type of school, and number of years teaching, did not independently predict the presence of emotional exhaustion symptoms in the participants.

### 3.3 Logistic regression to identify predictors of depersonalization/cynicism

Table 4 gives an overview of the binary logistic regression analysis to determine predictors of depersonalization. Fourteen predictors that had a significant association (*p* < 0.05) or near significant association (0.1 ≥ *p* ≥ 0.05) with depersonalization in the univariate analysis were included in a binary logistic regression model. The regression model was statistically significant; *Χ*^2^ (df = 44; *n* = 751) = 141.50 *p* = 0.000 < 0.0005, suggesting that the model distinguished between respondents who experienced depersonalization and those who did not. The model explained between 17.20% (Cox and Snell *R*^2^) and 25.80% (Nagelkerke *R*^2^) of the variance and accurately classified 80.80% of cases.

**Table 4:** Logistic regression model for depersonalization/cynicism.

Variables	*B*	*S.E*	Wald	df	Sig.	Odds ratio	95% C.I. for odds ratio	
							Lower	Upper
Age (Years)								
18–25			1.768	3	0.622			
26–40	0.470	0.597	0.619	1	0.431	1.599	0.496	5.154
41–60	0.167	0.655	0.065	1	0.799	1.182	0.327	4.267
61 and above	0.391	0.894	0.191	1	0.662	1.478	0.256	8.530
Sex at birth								
Male								
Female	−0.875	0.288	9.206	1	**0.002**	0.417	0.237	0.734
Ethnicity								
Indigenous			12.940	7	0.074			
African Descendants	−0.186	1.232	0.023	1	0.880	0.831	0.074	9.288
East Asian	0.098	0.949	0.011	1	0.918	1.103	0.172	7.088
Latino	−2.396	1.296	3.418	1	0.064	0.091	0.007	1.155
Middle Eastern	−0.991	1.338	0.548	1	0.459	0.371	0.027	5.112
South Asian	−0.421	0.990	0.181	1	0.670	0.656	0.094	4.569
Caucasian	−1.334	0.622	4.599	1	**0.032**	0.263	0.078	0.892
Other ethnicities	−2.436	1.059	5.295	1	**0.021**	0.088	0.011	0.697
Housing status								
Own home			1.970	2	0.373			
Renting	0.444	0.324	1.875	1	0.171	1.559	0.826	2.946
Live with family or friend	0.035	0.646	0.003	1	0.957	1.035	0.292	3.672
Relationship status								
Single			14.904	4	**0.005**			
Married	0.648	0.350	3.421	1	0.064	1.911	0.962	3.796
Common Law/Partnered	1.248	0.368	11.497	1	**0.001**	3.482	1.693	7.161
Separated/Divorced	0.640	0.517	1.535	1	0.215	1.897	0.689	5.225
Other	1.990	0.779	6.528	1	**0.011**	7.318	1.590	33.693
# of children								
No child			8.545	4	0.074			
One child	−0.246	0.327	0.567	1	0.452	0.782	0.412	1.484
Two children	−0.677	0.294	5.315	1	**0.021**	0.508	0.286	0.904
Three children	−0.028	0.394	0.005	1	0.944	0.972	0.449	2.107
Four or more children	−0.951	0.567	2.815	1	0.093	0.386	0.127	1.173
School Situated								
Urban Setting	0.454	0.219	4.277	1	**0.039**	1.574	1.024	2.421
Number of years teaching								
5 years or less			0.940	3	0.816			
10 years or less but more than 5 years	0.210	0.374	0.316	1	0.574	1.234	0.593	2.566
20 years or less but more than 10 years	0.059	0.380	0.024	1	0.877	1.061	0.504	2.235
More than 20 years.	0.275	0.449	0.375	1	0.541	1.316	0.546	3.173
Major Role								
Elementary teacher			3.825	5	0.575			
Junior high teacher	0.367	0.291	1.600	1	0.206	1.444	0.817	2.552
Senior high teacher	0.386	0.326	1.404	1	0.236	1.471	0.777	2.787
Support Staff	−0.135	0.554	0.060	1	0.807	0.874	0.295	2.585
Administrator	−0.209	0.430	0.236	1	0.627	0.812	0.350	1.884
Other	−0.194	0.393	0.243	1	0.622	0.824	0.381	1.780
Average number of students/pupils in the classes you teach.								
20 or less			3.531	2	0.171			
21–27	−0.083	0.275	0.092	1	0.762	0.920	0.537	1.578
28 or more	0.392	0.325	1.457	1	0.227	1.48	0.783	2.799
Area of teaching specialization								
English			12.922	6	**0.044**			
Mathematics	0.460	0.442	1.085	1	0.298	1.585	0.666	3.768
Sciences (Physics, Chemistry, Biology)	0.754	0.442	2.918	1	0.088	2.126	0.895	5.053
Arts (History, Geography, Social Studies etc.)	0.268	0.413	0.420	1	0.517	1.307	0.582	2.937
Music	1.594	0.553	8.290	1	**0.004**	4.921	1.663	14.561
Physical education	1.337	0.575	5.406	1	**0.020**	3.806	1.234	11.743
Other	0.347	0.309	1.260	1	0.262	1.415	0.772	2.596
Source of stress								
Workload			7.475	4	0.113			
Student Behavior	0.605	0.249	5.890	1	**0.015**	1.832	1.124	2.987
Class size	−0.331	0.494	0.447	1	0.504	0.719	0.273	1.893
Limited management support	0.155	0.363	0.182	1	0.670	1.167	0.573	2.379
Other	0.446	0.332	1.800	1	0.180	1.561	0.814	2.994
Stress								
High stress	0.935	0.221	17.931	1	**0.000**	2.548	1.653	3.928
Resilience								
Low resilience	0.481	0.212	5.159	1	**0.023**	1.618	1.068	2.449
Constant	−1.578	0.962	2.692	1	0.101	0.206		

Sex at birth, relationship status, where the school is situated, area of specialization, stress, and resilience independently predicted the presence of depersonalization among participants. After controlling for all other variables in the regression model, male participants were 2.40 times (OR = 2.40; 95% CI: 1.36–4.22) more likely to experience cynicism than their female counterparts. Also, participants who described their marital status as common law/partnered and other were, respectively, 3.48 times (OR = 3.48; 95% CI: 1.69–7.16) and 7.32 times (OR = 7.32; 95% CI: 1.59–33.69) more likely to experience depersonalization than respondents who were single. Furthermore, participants who worked in the urban setting were 1.57 times more likely to experience depersonalization than those in the rural setting (OR = 1.57; 95% CI: 1.024–2.42). Similarly, respondents whose area of specialization was music were 4.92 times (OR = 4.92; 95% CI: 1.66–14.56) and those who selected physical education were 3.81 times (OR = 3.81; 95% CI: 1.23–11.74) to experience depersonalization than those whose area of specialization was English. In addition, participants who experienced high-stress symptoms were 2.55 times (OR = 2.55; 95% CI: 1.65–3.93) more likely to experience depersonalization than those who did not. Furthermore, respondents with low resilience were 1.62 times (OR = 1.62; 95% CI: 1.07–2.45) more likely to experience depersonalization than those who did not.

Participants ethnicity as a variable did not make a unique statistically significant contribution to the model. However, participants who were Indigenous were, respectively, 3.80 times (OR = 3.80; 95% CI: 1.12–12.82) and 11.36 times (OR = 11.36; 95% CI: 1.43–90.91) more likely to present with depersonalization compared with Caucasians and those with “other” ethnic backgrounds when all other factors are controlled. Furthermore, the number of children as a variable did not make a unique statistically significant contribution to the model. However, participants who had no children were 1.97 times (OR = 1.97; 95% CI: 1.11–3.50) more likely to experience cynicism compared to those who had two children. Finally, the source of stress as a variable did not contribute significantly to the model, but respondents who reported student behavior as their source of stress were 1.83 times (OR = 1.83; 95% CI: 1.12–2.99) more likely to experience depersonalization than those who reported workload as source of stress.

Other demographic and professional variables, such as age, housing status, number of students in the class, and teaching experience, did not independently predict depersonalization or cynicism among participants.

### 3.4 Logistic regression to identify predictors of lack of professional fulfillment/accomplishment

Table 5 illustrates the binary logistic regression analysis for the predictors of lack of professional fulfilment or accomplishment. Five predictors that had a statistically significant association (*p* < 0.05) or near significant association (0.1 ≥ *p* ≥ 0.05) with lack of professional accomplishment on univariate analysis were included in a binary logistic regression model. The regression model was statistically significant; *Χ*^2^ (df = 13; *n* = 751) = 62.70 *p* = 0.000 < 0.0005, suggesting that the model could differentiate between participants who lacked personal fulfilment and those who did not. The model only explained between 8.00% (Cox and Snell *R*^2^) and 11.30% (Nagelkerke *R*^2^) of the variance and correctly classified 69.40% of cases. Stress and resilience as predictors of lack of professional accomplishment made the most significant unique contribution to the regression model and independently predicted the presence of a lack of professional accomplishment among participants. After controlling for all other variables in the regression model, participants who experienced high-stress symptoms were 2.34 times (OR = 2.34; 95% CI: 1.64–3.35) more likely to experience a lack of professional fulfilment than those with low stress. Similarly, respondents with low resilience were 1.73 times more likely to experience lack of professional fulfilment than those with high resilience (OR = 1.73; 95% CI: 1.23–2.43). Other demographic and professional variables, such as age, major role, and a number of years teaching, did not independently predict the presence of a lack of professional fulfilment in the participants.

**Table 5:** Logistic regression model for lack of professional fulfillment.

Variables	*B*	S.E	Wald	df	Sig.	Odds ratio	95% C.I. for odds ratio	
							Lower	Upper
Age (Years)								
18–25			2.086	3	0.555			
26–40	−0.539	0.480	1.261	1	0.261	0.583	0.228	1.494
41–60	−0.323	0.517	0.389	1	0.533	0.724	0.263	1.996
61 and above	−0.497	0.759	0.428	1	0.513	0.609	0.137	2.695
Number of years teaching								
5 years or less			5.296	3	0.151			
10 years or less but more than 5 years	0.119	0.303	0.156	1	0.693	1.127	0.622	2.040
20 years or less but more than 10 years	−0.246	0.297	0.686	1	0.408	0.782	0.437	1.400
More than 20 years.	−0.574	0.351	2.672	1	0.102	0.563	0.283	1.121
Major role								
Elementary teacher			2.854	5	0.723			
Junior high teacher	−0.133	0.229	0.337	1	0.562	0.875	0.559	1.372
Senior high teacher	0.143	0.241	0.355	1	0.552	1.154	0.720	1.850
Support Staff	0.026	0.423	0.004	1	0.950	1.027	0.448	2.353
Administrator	−0.339	0.349	0.944	1	0.331	0.713	0.360	1.412
Other	−0.315	0.321	0.967	1	0.325	0.730	0.389	1.368
Stress								
High stress	0.851	0.183	21.730	1	**0.000**	2.341	1.637	3.348
Resilience								
Low resilience	0.547	0.173	9.964	1	**0.002**	1.728	1.230	2.427
Constant	−0.598	0.430	1.931	1	0.165	0.550		

## 4 Discussion

The current study aimed to explore the prevalence and correlates of the three dimensions of burnout (emotional exhaustion, depersonalization, and lack of accomplishment) in the Canadian context, focusing on three participating provinces: Newfoundland and Labrador, Alberta, and Nova Scotia. Burnout has been presumed to start with the development of emotional exhaustion in response to overload and high demands, which then lead to detachment and negative reactions towards people and the job (Depersonalization or cynicism) and subsequently feelings of inadequacy and failure [reduced personal accomplishment or professional inefficacy if the status quo continues (1)]. The prevalence of emotional exhaustion, depersonalization, and lack of accomplishment among teachers in this study was 76.9, 23.2, and 30.8%, respectively, which are consistent with prevalence of burnout among teachers in other jurisdictions (ranging from 25.12 to 74%) reported in a scoping review (5). The prevalence reported in this study for all three burnout subscales was however higher than that reported in a systematic review and metanalysis of 12 studies which assessed burnout among [physical education teachers (12)]. The later review reported a prevalence of 28.6% for high emotional exhaustion, 14.5% for depersonalization and 29.5% low personal accomplishment among physical education teachers. A survey during the initial stages of the pandemic among Canadian teachers also reported increased emotional exhaustion and lack of accomplishment among school teachers (49). However, the prevalence rates reported in this study are higher than those reported in a study among nurses, which was 25% for high emotional exhaustion, 15% for depersonalization, and 22% for low personal accomplishment (50). The prevalence of emotional exhaustion reported in this study is also higher than what was reported in another study among teachers (11), where 49.6% of the teachers had moderate or severe emotional exhaustion. However, that study reported a comparatively higher prevalence of depersonalization, 28.7% and lack of accomplishment (54.1%) among the teachers than in this study. The high prevalence of emotional exhaustion in this study, compared to the prevalence of depersonalization and lack of professional accomplishment, highlights the need to curtail the high emotional exhaustion among the participants in the under-studied provinces, as burnout has been suggested to be initiated by emotional exhaustion (1). With respect to inter-provincial differences in prevalence for the dimensions of burnout, this study revealed statistical differences on chi square test only for emotional exhaustion among teachers in the three provinces, with Alberta recording the highest prevalence (83.1%), followed by Nova Scotia (77.5%) and Newfoundland and Labrador recorded the lowest prevalence (65.7%).These inter-provincial differences in prevalence for the burnout syndrome were however not statistically significant when other factors were controlled for in regression models. It is possible that other factors which were not assessed and included in the model, such as inter-provincial variations in conditions of service (annual salary ranges), and the number of years from the start of a teaching career before teachers reach the top salary (36) accounts for variations in prevalence that were noted on chi-square examination.

### 4.1 Predictors of emotional exhaustion

The challenges teachers face are diverse and teacher specific at different time points. Teachers’ appraisal of the extent of this challenge and its impact is therefore the determinant of teachers’ stress levels. In this case, resilience becomes an important element. Teacher resilience is the degree to which they are able to uphold a set of positive attributes about the profession despite a variety of demands, pressures, and challenges inherent in the work (51). Our results show that respondents with low resilience were 3.26 times more likely to experience emotional exhaustion symptoms than those who did not. Similar result was reported in another study which found resilience as a predictor of emotional exhaustion (52). Respondents with high stress symptoms were about seven times more likely to experience emotional exhaustion than those who did not. This outcome was expected as stress has often been linked to teacher burnout comprising of three main elements; attitudinal, physical and emotional exhaustion (53). A systematic review which examined the impact of organizational context on teacher burnout, reported that classroom disruption, perceived collective exhaustion, and work climate which are all associated with high stress exhibited statistically significant detrimental effects on emotional exhaustion (54).

The current study indicates that participants who worked in the urban setting were 1.63 times more likely to experience emotional exhaustion than those in rural settings. The literature suggests that urban school teachers experience significantly more stress than rural school teachers, and this may lead to burnout (55). Another review (56) noted that teachers in rural settings tend to teach relatively smaller class sizes, report satisfaction with their work environments and have relatively few problems with discipline which may contribute to the absence of emotional exhaustion. Our finding corroborates with this review finding, suggesting that teachers in urban settings experience more emotional exhaustion compared to rural teachers. Our study finding, however, is contrary to the finding from another study (11), which reported that emotional exhaustion is independent of residence (rural–urban) of participants.

In our current study, participants who had no child were 2.26 times and 3.53 times more likely to experience emotional exhaustion than those with three children and those with four or more children, respectively. The possible explanation is that teachers with no child are likely younger and have less teaching experience in comparison with those with children who may be older and have more teaching experience. On the contrary teacher’ school work is usually compounded by the demands at home of teachers and it would have been expected that teachers with three or more children would have experienced increased emotional exhaustion due to increased demand at school and home. Normally, the work of a teacher extends beyond the classroom to homes as teachers plan lessons, mark assessments, and perform other roles to progress the curriculum (57). Other demographic and professional variables, such as age, ethnicity, the school teachers teach and number of years teaching, did not independently predict the presence of emotional exhaustion symptoms in the participants. However, a study (14) has implicated age as a significant predictor of emotional exhaustion. This difference may be attributed to differences in the countries of research and other cultural differences not explored in the study. The number of years of teaching or teaching experience has normally been associated with educators’ ability to manage the class effectively, make accurate assumptions, and deliver the curriculum with precision. Beginner teachers have been suggested to navigate and articulate issues with more difficulty, leading to increased burnout (2). Our study did not, however, provide evidence for this assumption.

### 4.2 Predictors of depersonalization

Sex at birth, relationship status, where the school is situated, area of specialization, stress, and resilience independently predicted the presence of depersonalization among participants. Our results contradict findings from another study, which suggested that depersonalization was independent of gender, marital status, or residence (rural–urban) (55).

Being married, in a relationship or lack of support have been considered to reduce the risk of mental health issues and serves as protective factor against the same (58). Respondents who were married or separated did not show any statistically significant likelihood of developing depersonalization compared to the single participants. However, participants who described their marital status as common law/partnered and others were three and half times and 7.32 times, respectively, more likely to experience depersonalization compared to those who were single. One possible explanation is that teachers tend to work extra hours outside of the classroom, and this may be affecting the nature of common law/partnered and other relationships as these may be less stable than marriages. In addition, most teachers in one study (59) reported that occupational stress significantly impacted their personal relationships, which may explain the increased likelihood of the presence of depersonalization in participants who identified their marital status as common law/partnered. This study found no significant difference between single individuals and those who were divorced. On the contrary, published literature indicates that individuals who are single tend to experience higher burnout levels (2). In contrast, a meta-analytic study among nurses reported a statistically significant relation between depersonalization and marital status and gender, with males who are single or divorced experiencing more burnout (60). Some aspects of this finding support our study, whilst others are contradictory. Our study indicates, for instance, that male participants were about two and a half times more likely to experience cynicism than their female counterparts. Similar findings were reported in another study which indicated that emotional exhaustion and depersonalization were significantly lower in female teachers than in male teachers (61). Additionally, in a meta-analysis it was reported that men experienced more depersonalization than women, whilst women experience more emotional exhaustion than men (62). Another study (63) theorized that men are more likely to experience depersonalization than females due to greater work stress and inadequate coping skills. On the contrary, another study (64) suggested that females are more likely to experience burnout than men as they navigate their professional roles. Future study may explore which particular sex and gender experience depersonalization associated with number of children as the current study did not explore this.

Although number of children as a variable did not make a unique statistically significant contribution to the regression model, participants who had no child were about two times more likely to experience cynicism than those with two children. The finding corroborates finding from another study which reported that educators’ perceptions about burnout differed dependent on having a child or not. The study suggests that teachers who have children or have the responsibility of child rearing may be more tolerate with problems arising from work and life (3). Teachers with no child may also experience increased cynicism possibly because they are younger and inexperienced and could be struggling with the demands of the profession.

Our findings suggest that participants who worked in the urban setting were one and a half times more likely to experience depersonalization than those in rural settings. Our result is contrary to another study reporting that Depersonalization was independent of gender, marital status or school location (rural–urban) (55).

Similarly, respondents whose area of specialization was music and physical education were about five and four times, respectively, more likely to experience depersonalization than those whose area of specialization was English. It’s possible that the academic demands and stressors associated with music and physical education specializations are more intense compared to English. For example, music programs may involve rigorous practice schedules or performance expectations, while physical education programs may have demanding physical requirements. The heightened stress associated with these specializations could contribute to a higher likelihood of depersonalization. Moreover, music teachers have been reported to be substantially more burnt out than mathematics teachers in another study (65). Additionally, physical education classes are largely dependent on equipment and facilities and are mainly conducted outdoors in tracks, courts, school yards or gyms, which may increase problems for sustaining class discipline, leading to likely burnout (Depersonalization) (66). This may probably be the reason for the surge in depersonalization among this group.

In addition, participants who experience high stress symptoms were two and a half times more likely to experience depersonalization than those who did not. Stress has generally been associated with burnout and has been reported to be a significant predictor of burnout, fatigue and exhaustion (67). Our study is also consistent with another study which reported that acute stress disorder was significantly and positively associated with emotional exhaustion and depersonalization dimensions of job burnout (61). Furthermore, respondents with low resilience were about one and a half times more likely to experience depersonalization than those who did not. Consistent with our study, another study reported a positive relationship between emotional exhaustion and depersonalization and low levels of resilience (68).

Participants’ ethnicity as a variable did not make a unique statistically significant contribution to the model. However, participants who identified as indigenous were four and 11 times more likely to present with depersonalization compared with Caucasians and those who indicated other as their ethnicity, respectively. There may be likely cultural differences in the way individuals from different ethnic backgrounds perceive or cope with stressors, potentially influencing the likelihood of experiencing depersonalization. This is supported by a study outcome that reported both universal and culture-specific effects on burnout, signifying the need for a cross-cultural perspective to study burnout (69). However, other variables not included in the model could be relevant to understanding the relationship between ethnicity and depersonalization.

Finally, the source of stress as a variable did not contribute significantly to the model, but respondents who reported student behavior as their source of stress were about two times more likely to experience cynicism than those who reported workload as source of stress. Student behavior patterns have been reported to be a source of stress among teachers (20), and this is reiterated in our current study. Other demographic and professional variables, such as age, housing status, number of students in the class, major role, and teaching experience did not independently predict depersonalization among participants. On the contrary, one study has associated age with depersonalization (70). There’s a lack of literature in relation to number of students in the class and burnout. However, class size may have a direct relationship with class management and students’ behavior. Related to this, class size has been reported to significantly predict anxiety among teachers (14), which may encompass anxiety about class management.

### 4.3 Predictors of lack of professional accomplishment

Participants who experienced high stress symptoms were about two times more likely to experience lack of professional fulfillment than those with low stress. Our study finding is consistent with other studies (67, 71) among teachers which reported that occupational stress was a significant predictor of burnout. Similarly, respondents with low resilience were almost two times more likely to experience a lack of professional fulfilment than those with high resilience. Similar findings were reported in another study indicating that resilience significantly predicts personal accomplishment (14). Other demographic and professional variables, such as age, major role and teaching experience, did not independently predict depersonalization among participants. The current outcome is contrary to another study (2), which reported that among younger employees, the level of burnout is higher than it is among those over 30 or 40 years old. The study noted that teachers with increased teaching experience are less prone to burnout. Conflicting results were also reported in another study in terms of age, which indicated that depersonalization and low professional accomplishment were significantly associated with age (70).

### 4.4 Implications for policy and practice, and future research

This study has established that the prevalence of the three burnout syndromes among the three Canadian provinces range from 23.2 to 76.9%, and stress and low resilience were the key predictors of the burnout syndromes. Disparities in the prevalence of emotional exhaustion, a burnout dimension among teachers in the three participating provinces, highlight the importance of considering other contextual factors which were not assessed in this study but may contribute to stress, such as regional economic conditions, organizational cultures, and social support systems, in understanding the prevalence and manifestations of burnout. Research is necessary to explore the underlying mechanisms driving these provincial disparities and to develop strategies to mitigate burnout and enhance occupational health across provinces. School location was also a significant predictor of emotional exhaustion which may warrant further research into aspects of rural and urban settings causing burnout. The current study also sheds light on the complex relationship between resilience, stress, and burnout and reveals compelling insights into the predictors of burnout across all the various burnout dimensions. This suggests that individuals with higher levels of resilience are better equipped to withstand the demands and pressures of their roles, mitigating the risk of burnout. The findings underscore the importance of resilience and the need to build resilience among teachers while aiming to reduce stress. Thus, teachers will be better able to bounce back from setbacks, cultivate effective coping strategies, and maintain a sense of purpose and optimism, which will serve as a protective buffer against the toxic effects of chronic stress, hence occupational burnout. Conversely, stress as a significant predictor of burnout underscores the detrimental impact of stress on individuals’ physical, emotional, and cognitive wellbeing and highlights its role as a key precipitant of burnout. It is vital to address teacher’s burnout thereby empowering teachers to thrive in their roles. This will inadvertently be a gain for the students they teach. Administrators, school boards, organizations and policymakers can implement targeted interventions aimed at addressing systemic stressors, fostering resilience, and promoting a supportive work environment conducive to educators’ wellbeing.

### 4.5 Limitations

There are some limitations worth mentioning. First, the demographic variables in the study may not reflect the demographics of teachers in Canada, as only teachers in three provinces were involved in the study. Again, our study participants were predominantly females, 87.7%, and only 12.3% male participants thus, the study findings may not be generalizable. Secondly, the scales used to assess burnout although standardized are not meant to be diagnostic. Thirdly, the response rate for our study is low, although it is common for web-based survey completion rates, which typically yield lower response rates than paper-based surveys (72, 73). Despite these limitations, our study is the first to explore the prevalence and independent predictors of the three burnout dimensions in the three Canadian provinces, and our findings would be of interest to policy makers, particularly those working in the education sector.

## 5 Conclusion

There was a high prevalence of emotional exhaustion, depersonalization and lack of accomplishment among teachers in this study, with high stress and low resilience being key predictors of the three burnout syndromes. The cumulative burden of stress undermines individuals’ resilience and erodes their capacity to sustain engagement and fulfillment in their professional roles. By identifying resilience and stress as central predictors of burnout across the three burnout dimensions, the study highlights the importance of schools deploying interventions aimed at enhancing resilience, mitigating stress, and fostering a supportive work environment conducive to employee wellbeing.

## Data availability statement

The raw data supporting the conclusions of this article will be made available by the authors, without undue reservation.

## Ethics statement

The studies involving humans were approved by University of Alberta Ethics Review Board (Pro00117558) and the Dalhousie University Human Research Ethics Review Board (REB # 2023-6840). The studies were conducted in accordance with the local legislation and institutional requirements. The ethics committee/institutional review board waived the requirement of written informed consent for participation from the participants or the participants’ legal guardians/next of kin because this is an anonymous online survey and so written informed consent will not have been possible. The ethics board approved implied consent when participants complete and return the online survey. All participants were provided with information leaflets about the study and were made aware that consent will be implied if they complete and return the survey.

## Author contributions

BA: Conceptualization, Data curation, Formal analysis, Investigation, Methodology, Project administration, Software, Validation, Writing – original draft, Writing – review & editing. RL: Software, Validation, Writing – review & editing. YW: Software, Supervision, Validation, Writing – review & editing. VA: Conceptualization, Data curation, Funding acquisition, Investigation, Methodology, Project administration, Resources, Software, Validation, Writing – review & editing.
